# Protocol to investigate teacher-child neural scaffolding in executive functions and school readiness development in preschool children

**DOI:** 10.1371/journal.pone.0346921

**Published:** 2026-08-25

**Authors:** Rachel Lara Green, Choon San Kevin Wee, Vaidehi Ramanarayanan, Haoxuan Zhang, Teo Ling Zheng, Xinchen Fu, Timurcan Ozcelik, Angshuk Dutta, Jennifer Ruth Dulimar, Vanessa Reindl, Kerstin Konrad, Kenneth Poon, Victoria Leong

**Affiliations:** 1 Early Mental Potential and Wellbeing Research (EMPOWER) Centre, Nanyang Technological University, Singapore, Singapore; 2 Psychology, School of Social Sciences, Nanyang Technological University, Singapore, Republic of Singapore; 3 Cambridge-NTU centre for Lifelong Learning and Individualised Cognition (CLIC) Nanyang Technological University, Singapore, Singapore; 4 Institute of Medical Psychology and Medical Sociology, Uniklinik RWTH Aachen, Aachen, Germany; 5 Institute of Neuroscience and Medicine, Translational Neuroscience (INM-10), Forschungszentrum Jülich, Jülich, Germany; 6 National Institute of Education, Nanyang Technological University, Singapore, Singapore; PLOS: Public Library of Science, UNITED KINGDOM OF GREAT BRITAIN AND NORTHERN IRELAND

## Abstract

The transition to primary school is a key milestone in a child’s life and how ready a child is for this change can affect their later academic success. Executive functions are regarded as an important contributor to school readiness, and the early development of executive functions are strongly moderated by the child’s social context, such as caregiver relationships. Whilst the role of parent-child relationships on the development of executive functions is well established, less is known about the role of other caregivers, such as teachers. A series of novel teacher-child electroencephalography hyperscanning interaction tasks have been developed to explore how teachers scaffold preschool children’s emerging literacy skills, problem solving and executive functions. Behavioural and neural synchrony will be measured during interactive tasks. Children’s school readiness will be measured, as well as teacher and child executive functions and intelligence. The relative contribution of the teacher and child’s individual characteristics to behavioural neural synchrony will be explored, as well as the effects teacher scaffolding style. These insights are necessary to ensure the quality of infant care and preschool in a climate where attendance is becoming increasingly popular worldwide.

## Introduction

A successful transition to primary school is an important milestone in a child’s life, which places increasing demands on their executive functions (EFs). EFs are cognitive processes involved in the control and coordination of information which assists goal-directed behaviour [1]. A child might use EFs to remember and follow the teacher’s instructions, complete tasks independently and to smoothly transition between tasks, as well as to stay on task and ignore distractions. There are individual differences in how ready children are to learn in structured environments and EFs have been reported to be the single best predictor of school readiness [2,3]. This highlights the importance of EFs in child development and the importance of understanding which factors promote development of EFs in preschool children.

Interaction between teachers (also referred to as educators or caregivers in some contexts) and children (or students) are thought to be an important factor in the development of preschool children’s EFs. This is supported by research reporting the efficacy of using story books and puzzles to scaffold the development of children’s EFs [4]. Howard and colleagues reported that storybook reading improved preschool children’ s working memory and shifting abilities [4]. Furthermore, links between teacher-child relationships and academic performance have been reported in primary school children [5,6]. A previous meta-analysis of 23 independent samples, which were predominantly adults, reported that interpersonal neural synchrony was positively associated with learning outcomes [7]. Interpersonal neural synchrony is defined as correlation or coherence in time, space and/or frequency dimensions between two communicating brain signals [7,8]. It is unclear exactly *how* preschool teachers support development of children’s EFs; therefore, the current investigation will explore the potential involvement of neural synchrony in teacher-child interactions that support development of EFs.

### School readiness and executive functions

The age children start school varies across countries, but in Singapore children begin primary school around age 6 or 7 years. Prior to this, it is increasingly common for Singaporean children to attend infant care and preschool, as is also true in many countries worldwide. This presents an invaluable opportunity to not only prepare children for primary education, but also to potentially reduce gaps among children from disadvantaged home environments. Schools place increasing demands and expectations on children and require them to engage in formal structured learning activities. It is important to ascertain when children are ready for this next step. School readiness is a multidimensional construct that comprises cognitive, language, social-emotional-behavioural, physical and learning skills that enable children to learn and thrive in a school [9,10]. School readiness has been measured in terms of the understanding of basic concepts including colours, letters, numbers, shapes and social-emotional competence [11], as well as in terms of (pre-)academic abilities including (emerging) literacy skills and (emerging) mathematical knowledge [12]. Children vary in their school readiness and the extent to which children are ready for school when they start schooling has significant impacts on their social and academic achievement trajectories [13–17].

EFs are a constellation of partially dissociable higher-order cognitive processes that allow goal-directed behaviour. Significant development of EFs occurs between the ages of 3 and 5 years [18], which is the age which many children attend preschool. EFs play a significant role in the ability to regulate attention, emotion and behaviour in preschool children, which are essential for successful engagement in the classroom [19]. EFs provide a foundation for academic achievement and school performance by contributing to the children’s social functioning, emotion regulation and intentional learning [20]. This is exemplified by evidence that EFs are associated with children’s academic trajectories [13,21,22]. Cameron and colleagues report evidence for co-development of EFs and academic skills with improvements in EFs being associated with improvements in applied problem solving in 5 year old children, EF sub-domains were not assessed separately in this investigation [21]. Furthermore, EFs have long-term effects impacting several domains including academic success, impulse control, constraint and emotion regulation [23]. Previous intervention research indicates that EFs can be malleable and training of EFs has been linked to early academic success [13,24,25]. This highlights the critical role of EFs in school readiness and wider child development, as well as the importance of identifying factors that support development of EFs and the mechanisms by which this development occurs.

### Teacher scaffolding and interpersonal synchrony

Preschool teachers have a central role in their class’s preschool experience and development. Vygotsky’s [26] sociocultural theory dictates that learning is a social activity that occurs most effectively in social interaction with others. Additionally, children’s learning and development are optimally supported by scaffolding or assistance from more competent individuals [26]. Teachers may scaffold children’s learning by temporarily providing support to the child to allow them to complete a task that they would not be able to complete independently. As the child’s skill level increases, the teacher may gradually withdraw their support as the child becomes more capable until the child can complete the new task independently [27].

Scaffolding has been researched in the context of storybook reading (also referred to as read aloud; see 28) due to the frequency of this activity in preschools [28]. Storybook reading has a predictable, routinised format in which teachers can explore various texts with children, which has been reported to scaffold children’s emerging literacy skills [29]. Dialogic reading is an example of a common scaffolding technique employed during storybook reading contexts. This involves collaborative storytelling, in which the reader identifies and poses problems to the child and then scaffolds their answers [4,30]. Children’s participation in storybook reading sessions has been associated with positive effects of vocabulary growth [31–33] and comprehension [34,35], as well as giving children the opportunity to have more sophisticated conversations, going beyond perceptual understanding of stories and considering conceptual features [36]. Like EFs, children vary widely in their emergent literacy skills. Upon starting school many children know the alphabet, whereas many others do not [37]. To understand *how* children acquire literacy skills from their teacher, a dyadic storybook reading task has been developed to explore how preschool teachers scaffold children’s novel word learning and story comprehension.

One mechanism thought to influence exchange of knowledge between teacher and student is interpersonal neural synchrony*.* It has been theorised that this provides a neural underpinning for interpersonal exchange [38]. Interpersonal synchrony has been suggested to facilitate internal predictions about the self and predictions about the behaviour of others thereby optimising interactions [39]. Neuro-behavioural synchrony is posited to be an optimal learning environment for a child [40]; therefore, the present study aims to explore the contributions of interpersonal synchrony between teacher and child to the child’s learning outcomes.

Neural synchrony has been studied in preschool-age children previously using problem solving tasks. For example, Nguyen and colleagues [41] compared neural synchrony between preschool-age children and their parents when completing Tangram puzzles cooperatively, or independently. Unlike storybook reading, puzzle solving does not have an inherent rhythmicity making it an interesting context in which to study synchrony as synchrony is less likely to be masked by the rhythmicity of the task. Neural synchrony was greater under cooperative conditions and was associated with improved problem-solving performance [41]. Neural synchronisation was reported in temporal brain areas, which have been associated with effective social communication and mentalizing [42,43], as well as frontal areas, which have been previously associated with interactive decision making and EFs [42,43]. This highlights the relationship between EFs and their possible role in establishing neural synchrony or vice versa. The current investigation will use a range of teacher-child interaction tasks to assess the relative contribution of interpersonal neural synchrony to novel word learning and problem solving [44].

### The current investigation

This study will assess the relationship between teacher-child neural synchrony, child development of EFs and school readiness in preschool-age children. Children will be assessed cross-sectionally between the ages of 2 and 7 years. Children will complete EFs, vocabulary and school readiness assessments. Additionally, children and teachers will take part in interactive tasks whilst undergoing dyadic electroencephalography (EEG) and electrocardiogram (ECG) recordings to assess teacher-child interpersonal neural synchrony.

Study objectives and research questions:

1. Which teacher-child behavioural interaction characteristics are associated with individual differences in preschool children’s executive function and school readiness?
2. Does teacher-child neural synchrony during scaffolded tasks predict learning outcomes, including executive function and school readiness?

Explorative analyses:

1. Do different forms of teacher scaffolding (e.g., patterns of multimodal ostensive signals or dialogic reading framework) relate differentially to behavioural or neural synchrony?
2. How do teacher individual characteristics (e.g., EF, stress, temperament) moderate the relationship between scaffolding and child outcomes?

The study protocol will be described in eight main sections: study preparation, EFs tasks, school readiness assessment, vocabulary assessment, teacher-child interaction tasks, creative problem solving tasks, neurophysiological measures and questionnaire measures.

### Study preparation

#### Ethics.

This study has obtained ethical approval (IRB-2023-623 & IRB-2025-037) from the Nanyang Technological University Institutional Review Board (IRB). Data collection began in 5^th^ May 2025 and is planned to continue until 31^st^ September 2027 or when the recruitment target is reached. Written consent has been obtained from all adult participants and from the parents or guardians of child participants.

#### Recruitment overview.

Participants will be preschool children aged 2–7 years and previously unacquainted preschool teachers. The selected age range of 2–7 years reflects a critical developmental window during which core executive functions—such as working memory, cognitive flexibility, and inhibition—undergo rapid maturation [18,20]. This range also aligns with the typical age of preschool attendance in Singapore, where children begin preschool as early as age 2 or 3 years and transition to primary school at age 6 or 7 years [45]. Participants will be recruited via collaborations with preschool groups and independent participants. Children and teachers will be invited to the lab for 2 sessions lasting up to 3.5 hours each, ideally less than 2 weeks apart. Teachers will be invited to an additional individual assessment session lasting up to 3 hours. In this session teachers will complete individual EFs and intelligence assessments.

#### Sample size.

Research question 1 will be addressed using regression methods; therefore, a power calculation has been performed using RStudio (version 2024.04.1 + 748, Posit Software) and pwr library (version 1.30) to obtain an *R*_*2*_ value of 0.09, which is roughly equivalent to a medium effect as Cohen’s *d*. A sample of 187 children would permit the explanation of 9% of the variance with 12 predictor variables, 80% power and 5% chance of type 1 error. To account for attrition an additional 10% will be recruited to give a final sample size of 206 children. Up to 50 preschool teachers will be recruited, but fewer teachers may be required if they elect to take part with several children.

Children and teachers will be eligible if they do not have any identified genetic, metabolic, syndromic, or progressive neurological disorder, (e.g., epilepsy, Down or Rett Syndrome, Autism Spectrum Conditions, Attention Deficit Hyperactivity Disorder, Tuberous Sclerosis, Neurofibromatosis, Fragile X Syndrome,).

#### Executive functions tasks.

A key aim of this study is measuring preschool children and teacher’s EFs. Three key EF domains will be measured including cognitive flexibility, inhibition and working memory. Teacher and child EFs tasks with be delivered electronically on a touch-screen computer.

**Tasks for preschool children:** Children’s EFs will be measured using age-appropriate tasks, such as the Eight boxes and Day-Night tasks. These tasks have been developed, adapted and standardised for a Singapore population (data unpublished). Both tasks are completed on a touch screen computer and scored on their speed and accuracy.

During the *Eight boxes task*, children are presented with 2–5 fruits in different spatial arrangements in 8 boxes. After a specified time, the fruits are removed from the boxes and the child is asked to place the fruits in the previously presented arrangement. Children complete 10-trials. This task measures working memory and cognitive flexibility.

The *Day-Night task* uses similar principles as the Stroop [46] or Dimensional Change Card Sort tasks [47]. Children are taught to sort cards using one rule, then they are asked to sort cards using a second rule and finally, they were asked to sort the cards using a third rule which is a combination of rules 1 and 2. This task measures cognitive flexibility, working memory and inhibition. Although these tasks have previously been used with children of a 3–7 years, adaptations may still be required to ensure that they are appropriate for the current context.

**Tasks for teachers:** Teachers are asked to complete a longer battery of computerised EFs tasks with tasks targeting specific EF sub-domains. Selected previously established tasks are listed below:

- Cognitive Flexibility
  - Wisconsin Card sort task [48]
  - Intra-extra-dimensional shift [49]
- Inhibition
  - Stop-signal [50]
  - Stroop [46]
- Working memory
  - Backward digit span [50]
  - Spatial working memory [49].

#### School readiness assessment.

The Brigance Early Learning Screens (III) is an assessment designed to assess children’s development from birth to 7 years [51]. The assessment samples children’s skills in a broad range of areas, including fine and gross motor, articulation, expressive and receptive language, general knowledge, personal-social skills and pre-academic skills. It is intended to be used to identify a child’s strengths and weaknesses as a form of early psychoeducational assessment, and to identify children who would benefit from additional diagnostic testing or special services. The test is scored by assigning point values to each assessed skill. Total points are compared with cut-offs with established norms. The Early Learning Screens (III) were originally normed in US populations. The test is administered by a research assistant and takes a total duration of between 15–25 minutes.

### Vocabulary assessment

The Peabody Picture Vocabulary Test is a standardised norm-referenced measure of receptive vocabulary based on Standard American English [52]. Children hear spoken words and are asked to point to the corresponding picture. A version that has been adapted and normed for a Singapore population [53] will be used.

### Teacher-child interaction tasks

#### Cooperation task.

This task was adapted from Reindl and colleagues [54]. A teacher and child will manipulate their respective on-screen animal using a keypress to catch a ball together. They will be instructed to press their keys in-time to “catch the ball together”. If the difference between the teacher and child’s key presses falls below a cut-off, the dolphins will jump together. If the difference is above the cut off, the faster respondent’s dolphin will catch the ball alone. The threshold was set to T =  1+ 1/8 (RT1 + RT2), where RT1 and RT2 are the response times of the two participants. This task will be split into 2 phases: teaching phase and test phase. During the teaching phase, the teacher and child can talk to discuss the best strategy to achieve the highest possible score, whereas the teacher and child are asked to complete the test phase in complete silence. Both phases will consist of 20 trials. In the test phase, a point will be awarded when the pair catch the ball together and a point will be deducted on trials when only one player catches the ball. The pair will start with a score of 100. Participants will be seated side-by-side facing a computer monitor with a divider between them. The divider will prevent the teacher and child looking at each other’s hands and will allow them to see the other player’s face.

#### Storybook reading task.

In this storybook reading task, the teacher and child will read a digital storybook for approximately 4 min and 30 seconds, adapted from [55]. The story, *Sally’s Space Adventure*, was designed to expose children to novel object label mappings, with objects and labels were chosen from the Novel Object and Unusual Name (NOUN) database [56]. *Sally’s Space Adventure* follows a narrative of an astronaut travelling through space to find the four novel objects needed to fix a broken rocket. All novel objects are named three times during the story: once with a colourful backdrop, once in isolation with a black backdrop, and once later in the story with an assigned function (e.g., fixing the engine of the rocket). The order and combination of novel words and novel objects will be fully randomised across participants. Teachers will not be instructed on how to engage with the child to preserve the natural dynamics of their student-teacher interactions while reading.

Children’s learning and retention of story information during the shared book reading will be assessed using a narrative recall exercise and a short quiz delivered by the teacher immediately after the story reading has finished.

#### Creative problem solving tasks.

1. Puzzle completion activities

Children will also be invited to participate in a series of Tangram puzzle tasks, where they will be seated next to the teacher. Children will be instructed to use a set of wooden puzzle pieces to complete a composite outlined picture on an indented board. There are a total of 4 unique puzzle task sets, separated into 4 phases. This includes a practice phase followed by 3 test phases. During the test phases only, the puzzle outlines provided to the student during the test phases include ‘shaded areas’ which require the combination of 2 or 3 separate pieces to successfully solve the puzzle. During the practice phase, the teacher will demonstrate to the student the correct way to use the provided puzzle pieces to complete the outlined picture. Supervision is provided by the teacher as needed and there is no time limit.

During each test puzzle, a new puzzle outline is provided to the student, and they are told that the puzzle contains shaded areas that may require them to do something different (compared to the non-shaded spaces). During the *first test phase*, the teacher does not provide feedback and encourages the child to complete the test as independently as possible. In the *second test phase*, the teacher actively provides verbal feedback as the child attempts to complete the puzzle. Teachers are instructed to use the teaching pedagogy with which they felt most comfortable. During the *third test phase*, children are asked to complete the puzzle independently with minimal guidance from the teacher, to assess the skills they have gained.

For each phase, the child’s completion time, and number of pieces in the correct position will be used to score their performance.

1. Picture Construction Activity

Children will be instructed to create a picture using the seemingly meaningless Tangram puzzle shapes. They will have 5 minutes to complete their picture. Upon completion they will be asked to describe the picture and give it a title. This task is scored on originality and elaboration (Torrance, 1964; but see Alabbasi et al., 2022).

1. Repeated Figures Activity

In this task, children will be asked to “build a path” to allow a character to get from one location to another. They will be presented with two squares in predefined positions attached to a laminated sheet. They will also be given 9 identical squares to use to connect those attached to the laminated sheet. Each completed solution will be reset after each response and the child was asked to try and create a new alternative solution. Feedback will be provided if their path isn’t compliant with the rules of the task. Children will have 4 minutes to play this game and the time taken to reset the pieces between solutions doesn’t contribute to the time. This activity was designed to elicit the tendency to repeatedly return to the same stimulus and perceive it in new ways. Scoring for this activity focused on fluency, and originality (Cramond, 1994).

### Neurophysiological measures

Whilst performing the teacher-child interaction tasks including the cooperation task, storybook reading task and creative problem tasks, as well as the EFs tasks, teachers and children will be asked to undergo concurrent EEG and ECG recordings. The EEG will be recorded using 32-channel system in a standard international 10/20 configuration. Event triggers will be sent to the EEG amplifiers to ensure synchronisation with task stimuli.

### Questionnaire measures

We will also administer teacher and caregiver self-report questionnaires, as well as caregiver-report questionnaires covering temperament, child behavior, parenting and family background information, such as their home language environment, parental education, household income etc.

#### Teacher-student relationship ratings.

The student- and teacher-report versions of the *Teacher-Student Relationship Inventory (S- & T-TRSI; 55)* will be used as a culturally adapted instrument to measure children’s and teacher’s subjective rating of each other. The TSRI (student and teacher versions) measure three factors: satisfaction, instrumental help, and conflict using a simple pictorial Likert scale. The psychometric properties of the TSRI have been validated for use in Singaporean populations using data from the Singapore Ministry of Education, showing reliability and validity across different genders, grade levels, and academic performance groups [57,58]. This student version of the questionnaire has been adapted for the slightly younger age of the children in this study. The student version will be administered by an experimenter to account for differences in reading ability.

#### Parent and teacher self-report questionnaires.

*Behavior Rating Inventory of Executive Function–Adult Version* (BRIEF-A) [59]: Self-report questionnaire designed to evaluate executive functions in adults aged 18–90 years, covering aspects such as inhibitory control, shifting, task monitoring, emotional control, and working memory.

*Adult Temperament Questionnaire* (ATQ) [60]: Self-report questionnaire designed to measure temperament traits in adults, focusing on dimensions such as effortful control, negative affect, extraversion, and orienting sensitivity.

*Perceived Stress Scale* (PSS) [61]: Self-report questionnaire that measures the perception of stress, evaluating how unpredictable, uncontrollable, and overwhelming respondents find their lives.

*State-Trait Anxiety Inventory* (STAI) [62]: Self-report measure that assesses both state anxiety (temporary condition) and trait anxiety (general tendency), providing insights into anxiety levels in individuals.

*Beck Depression Inventory* (BDI) [63]: Self-report questionnaire used to assess the severity of depression symptoms in individuals, covering emotional, cognitive, and physical aspects.

#### Parental questionnaires completed on behalf of their child.

*Behavior Rating Inventory of Executive Function – Preschool* (BRIEF-P) [64] or *The Behavior Rating Inventory of Executive Function* (BRIEF) [65] are parental report questionnaires designed to evaluate executive functions in children aged 2–5 and 5–18 years respectively. These assessments cover areas such as inhibition, working memory, and emotional control.

*Early Childhood Behavior Questionnaire* (ECBQ) [66] or *Childhood Behavior Questionnaire* (CBQ) [67,68]: Parent-report measures that assesses temperament in children aged 1.3–3 and 3–7 years respectively, focusing on dimensions like emotional reactivity, self-regulation, and sociability.

*Child Behavior Checklist* (CBCL) – versions for 1.5–5 and 6–18 years [69,70]: A widely used parent-report questionnaire that evaluates behavioral and emotional problems in children, covering areas like anxiety, depression, aggression, and social problems.

*Language Exposure Assessment Tool* (LEAT) [71]: A parent-report tool designed to assess the amount and type of language exposure a child receives, particularly in bilingual or multilingual contexts.

*Children’s Sleep Habits Questionnaire* – short form (CSHQ-sf) [72]: Parent-report questionnaire that evaluates sleep behaviors and problems in children aged 2–10 years, covering domains such as bedtime resistance, sleep duration, and night wakings.

*Alabama Parenting Questionnaire* – Preschool Revision (APQ-PR) [73] or *Alabama Parenting Questionnaire* (APQ) [74]: Parent-report measures that assesses parenting practices in children 2–6 and 6–18 years respectively, focusing on aspects like involvement, positive discipline, and inconsistent discipline.

*Family Interview for Genetic Studies* (FIGS) [75]: a series of questions design to obtain a comprehensive family history information on psychiatric disorders, medical conditions, and social functioning to aid in genetic research.

### Analysis plan

A combination of standard regression and machine learning methods will be used to identify characteristics of teacher-child social interactions that significantly predict EFs and school readiness scores (Research Question 1). Examples of behavioural indices of interaction quality are ostensive signals (gaze, speech, gestures), number of teacher prompts used, difficulty level of teacher prompts, number of prompts responded to by the child and complexity of child response. These behavioural indices of interaction quality along with individual teacher and child characteristics, such as personality or temperament, will be used to as factors to predict child EFs and school readiness. Data reduction methods such as least partial squares or principal components analysis may be used to reduce the dimensionality of data and prevent multicollinearity issues. These reduced factors will be used to predict school readiness and EFs.

To explore which characteristics of teacher-child neural coupling are associated with successful teacher-child interactions (Research Question 2) indices of neural coupling will be calculated from the dyadic EEG data and will be analysed in relation to learning outcomes on scaffolding tasks, as well as the behavioural indices of interaction quality that explain the most variance in EFs and school readiness. Examples of indices of neural coupling are weighted phase lag index (wPLI) or partial directed coherence (PDC). wPLI is a non-directional measure of neural coupling which will describe linear dependencies between the phase neural oscillations between two channels. This may be used to measure connectivity within the teacher or child’s brain, as well as between the teacher and child’s brains. PDC, alternatively, is a directed measure of connectivity and will provide additional information about the directionality of the flow of information. We will also explore the extent to which synchrony in the participants ECG is associated with successful teacher-child interactions.

For data-driven analyses, the combination of dyadic EEG and behavioural interaction data will be used to create multimodal AI models to phenotype children who are at risk of poorer school readiness outcomes. Specifically, we intend to utilise models such as cumulative link [76] and ordinal temporal deep learning models [77] to predict school readiness and executive functions. The use of neurophysiological recordings alone, as well as a combination of both neurophysiology and behavioural data mentioned in previous sections will be explored. Such models will be trained using mean squared error and likelihood-based objective functions to ensure best model fit [78]. The use of such models would also allow latent state analysis since both these models effectively learn low-dimensional features from high-dimensional neurophysiological and behavioural data relevant to executive functions and school readiness leading to mechanistic insights.

### Significance and impact

An ever-growing number of children are attending infant care and preschool worldwide, the quality of early education programmes varies considerably across providers, and neuro-scientifically validated teaching approaches are lacking. This project will explore empirical brain-based evidence on how early learning and EF development can be best supported by teacher-child interactions before entering primary school. Since good quality teacher-child interaction is likely to produce positive effects irrespective of the child’s intelligence and socio-economic status, this project may provide insights to assist in the development of policies to reduce economic-based school achievement gaps.

This project will explore the relationship between EFs, school readiness and teacher-student interaction. School readiness is particularly important and challenging for children with social and behavioural difficulties; individual differences in EFs are related to variations in school readiness in these children. A greater understanding of the interplay between EFs, school readiness and teacher-student interaction may stimulate the development of target interventions, as well as contribute to the development of new neuroscience-based metrics of teaching quality that could be useful in teacher training contexts.

Traditionally, teacher-child interactions are measured using behavioural observations or teacher reports, yielding modest to moderate effect sizes. While behavioural observations are often regarded as a gold standard, being more objective than self-reports, they come with several methodological disadvantages, such as being time-consuming and vulnerable to observer bias. This study will leverage on recent advances in hyperscanning and statistical analysis to adopt a sophisticated, sensitive and objective measure of teacher-child social interaction quality and success, based on dyadic brain-imaging measures. The combination of brain-imaging indices with traditional measures of social interaction quality will permit a deeper, more nuanced and precise understanding of the factors and neural mechanisms that underpin successful teacher-child interactions, and how these stimulate children’s early development of EFs.

### Limitations and future directions

The interactive tasks were developed to encourage the naturalistic dynamics of teacher-student interactions, which are likely to vary between teacher-student dyads, to increase the ecological validity and generalisability of the results. This is, however, at the sacrifice of experimental control. It is acknowledged that different scaffolding styles or approaches are likely to affect scaffolding effectiveness, but as scaffolding style or approach will not be experimentally manipulated here, the study is not optimised to detect such differences. Future research could seek to experimentally vary teacher scaffolding style or approach.

The current investigation will focus on EFs, school readiness and teacher-student interactions in typically developing children, but there are an ever-growing number of neurodevelopmental risk factors and disorders that put preschool children at-risk for poorer developmental outcomes. Future research should seek to explore the relationship between EFs, school readiness and teacher-student interactions in groups of children with neurodevelopmental risk factors and disorders.

While the study is conducted in Singapore and findings may reflect cultural and contextual factors unique to this setting, Singapore’s diverse, multilingual, and multicultural society makes it a compelling model for investigating early education practices in highly developed, globally connected contexts; nonetheless, cross-cultural replication will be important to assess generalisability.
